# Identification of a novel role for TL1A/DR3 deficiency in acute respiratory distress syndrome that exacerbates alveolar epithelial disruption

**DOI:** 10.1186/s12931-023-02488-1

**Published:** 2023-07-11

**Authors:** Dong Zhang, Jianning Zhang, Jintao Zhang, Xiang Ji, Qian Qi, Jiawei Xu, Yun Pan, Xiaofei Liu, Fang Sun, Rong Zeng, Liang Dong

**Affiliations:** 1 Department of Respiratory, Shandong Provincial Qianfoshan Hospital, Shandong University, Jinan, 250021 Shandong China; 2 Department of Respiratory and Intensive Care Unit, The First Affiliated Hospital of Shandong First Medical University and Shandong Provincial Qianfoshan Hospital, Shandong Institute of Respiratory Diseases, Jinan, China

**Keywords:** Alveolar epithelial barrier, TL1A/DR3 axis, Cathepsin E, Syndecan-1, Tight junction-associated zonula occludens 3

## Abstract

Alveolar epithelial barrier is a potential therapeutic target for acute respiratory distress syndrome (ARDS). However, an effective intervention against alveolar epithelial barrier has not been developed. Here, based on single-cell RNA and mRNA sequencing results, death receptor 3 (DR3) and its only known ligand tumor necrosis factor ligand-associated molecule 1A (TL1A) were significantly reduced in epithelium from an ARDS mice and cell models. The apparent reduction in the TL1A/DR3 axis in lungs from septic-ARDS patients was correlated with the severity of the disease. The examination of knockout (KO) and alveolar epithelium conditional KO (CKO) mice showed that TL1A deficiency exacerbated alveolar inflammation and permeability in lipopolysaccharide (LPS)-induced ARDS. Mechanistically, TL1A deficiency decreased glycocalyx syndecan-1 and tight junction-associated zonula occludens 3 by increasing cathepsin E level for strengthening cell-to-cell permeability. Additionally, DR3 deletion aggravated barrier dysfunction and pulmonary edema in LPS-induced ARDS through the above mechanisms based on the analyses of DR3 CKO mice and DR3 overexpression cells. Therefore, the TL1A/DR3 axis has a potential value as a key therapeutic signaling for the protection of alveolar epithelial barrier.

**Supplementary Information:**

The online version contains supplementary material available at 10.1186/s12931-023-02488-1.

## Introduction

Acute respiratory distress syndrome (ARDS) is an acute respiratory disease that involves blood–air barrier dysfunction. ARDS is mainly manifested as pulmonary edema caused by hyperpermeability of the vascular endothelium and alveolar epithelium [1]. A large number of drugs have been developed to improve the blood–air barrier for the clinical treatment of ARDS, but some patients still exhibit poor response to treatments. The COVID-19 pandemic has led to an increase in the incidence and mortality of ARDS, and the lack of effective treatments in severe cases is a major issue [2]. This condition further highlights the importance of understanding the mechanism underlying alveolar epithelial barrier function, which may provide a basis for the development of new strategies for ARDS treatment.

The alveolar epithelium barrier is mainly composed of the cell surface glycocalyx and intercellular tight junctions. The glycocalyx, a complex layer of polysaccharides and proteins located on the surface of the epithelium, is composed of core protein and glycosaminoglycan chains and is rich in syndecan-1 (SDC-1) [3]. SDC-1 is essential for inflammatory response, immune disorders, structural remodeling, hyperpermeability, and other pathophysiological processes [4–6]. Tight junctions are mainly composed of three integral membrane proteins (occludins, claudins, and junctional adhesion molecules) and peripheral cytoplasmic proteins such as zonula occludens proteins (junction-associated zonula occludens [Tjp]-1, Tjp-2, and Tjp-3), which participate in information transmission, cellular transport, cell proliferation, and differentiation [7, 8]. Proteases such as matrix metalloproteinases (MMPs) and heparanase (HPA) mediate damage to the glycocalyx and tight junction structures, leading to cell-to-cell hyperpermeability [9–11]. In addition, members of the cathepsin family, a class of lysosomal proteases activated in acidic environments, function in protein degradation and cytokine regulation [12]. The role of cathepsin E (Ctse) has been a focus of recent research on proteases [13, 14]. However, the role of Ctse in ARDS has not been clearly determined.

The TNF superfamily is large, and it includes TNF ligands (*n* = 19) and receptors (*n* = 29), as determined based on large-scale sequencing of the human and mouse genomes. These members function in immune cells, as well as in respiratory and intestinal diseases, and some members may act as a double-edged sword. Tumor necrosis factor-like cytokine 1A (TL1A, also known as *TNFSF15*) is the only known death receptor 3 (DR3, also known as *TNFRSF25*) ligand [15]. The TL1A/DR3 axis plays a role in the regulation of intestinal immunity and fibrosis, asthma airway remodeling, and other autoimmune and inflammatory diseases that aid in exacerbating disease progression [16–18]. However, some researchers have proposed that the TL1A/DR3 axis has a protective role in some disease models. A novel role for TL1A/DR3 in protection against intestinal injury was reported by Jia et al. [19]. Yang et al. revealed the protective effect of TL1A against intracerebral hemorrhage-induced secondary brain injury and infection [20]. In addition, Li et al. confirmed that TL1A maintains the blood–retina barrier by modulating SHP-1-Src-VE-cadherin signaling in diabetic retinopathy [21]. However, the role of TL1A/DR3 in ARDS has not been explored.

In the present study, TL1A knockout (KO) or alveolar epithelium conditional KO (CKO), and DR3 CKO mice were constructed to explore the mechanism of action of TL1A/DR3 in the alveolar epithelium in ARDS. The results provide the first evidence for the protective effect of the TL1A/DR3 axis on ARDS.

## Materials and methods

### Reagents

Lipopolysaccharide (LPS) from *Escherichia coli* 055:B5 (L2880), 4, 6-diamino-2-phenylindole dihydrochloride (DAPI, 28718-90-3), and fluoresceine isothiocyanate (FITC)-dextran (FD4, 60842-46-8) were obtained from Sigma-Aldrich (St. Louis, MO, USA). Anti-SDC-1 (ET1703-42), anti-Tjp-3 (ER61305), anti-TL1A (ER1917-90), anti-Ctse (ER62913), anti-DR3 (ER65462), anti-GAPDH (ET1601-4), and HRP-conjugated goat anti-rabbit IgG (HA1001) were obtained from HUABIO (Zhejiang, China). Anti-FITC (ab19224), anti-prosurfactant protein C (SP-C, ab90716), anti-SDC-1 (ab128936), recombinant human MMP9 (ab168863), and Ctse activity assay kit (ab211081) were obtained from Abcam (Cambridge, UK). Anti-DR3 (PA5-19882) and anti-Tjp-3 (#36-4100) were obtained from Thermo Scientific (Waltham, US). Recombinant human active heparanase/HPA protein (7570-GH) and Ctse protein (1294-AS) were obtained from RD Biotechnology Ltd. (Franche-Comte, France). The BCA protein assay kit (PC0020), hematoxylin–eosin (HE) stain kit (G1120), and broad spectrum SP kit (SP0041) were sourced from Solarbio (Beijing, China). Anti-Tjp-3 (#3704) was obtained from Cell Signal Technology (Danvers, MA, USA). Dylight 488 goat anti-mouse IgG (A23210) and goat anti-rabbit IgG (A23220), Dylight 549 goat anti-mouse IgG (A23310), and goat anti-rabbit IgG (A23320) were sourced from Abbkine (Wuhan, China).

### Human subjects

Human subjects were diagnosed with ARDS based on the ARDS Clinical Practice Guideline 2021. The human specimens were screened in the Department of Pathology, Department of Respiratory and Intensive Care Unit of The First Affiliated Hospital of Shandong First Medical University and Shandong Provincial Qianfoshan Hospital according to the diagnostic criteria for ARDS and negative control criteria. This study was approved by the Human Research Ethics Committee of The First Affiliated Hospital of Shandong First Medical University and Shandong Provincial Qianfoshan Hospital.

### MLE-12 and hPAEPIC culture and treatment

Mouse alveolar epithelial cells (MLE-12, ZQ0470) and cell complete medium (ZQ-601) were sourced from Shanghai Zhong Qiao Xin Zhou Biotechnology. Human pulmonary alveolar epithelial cells (hPAEPICs, HUM-iCell-a002) and the primary epithelial cell culture system (PriMed-iCell-001) were obtained from iCell Bioscience (Shanghai, China).

MLE-12 and hPAEPICs were cultured in six-well culture plates at 37 °C in an incubator with 95% air and 5% CO_2_. MLE-12 and hPAEPICs were stimulated using 10 μg/mL LPS for 2, 4, and 6 h [22, 23]. Western blot analysis revealed that the expression levels of the TL1A/DR3 axis remarkably decreased at 6 h after LPS stimulation. Therefore, LPS stimulation for 6 h was selected as the appropriate time point for hPAEPICs.hPAEPICs were divided into four groups to explore the effects of TL1A: (1) CTL group as a negative control, (2) Lentiviruses 6 (LV6)-TL1A group, in which hPAEPICs were transfected with LV6-TL1A for TL1A overexpression (OE), (3) LPS group, in which hPAEPICs were stimulated by 10 μg/mL LPS for 6 h, and (4) LV6-TL1A + LPS, in which hPAEPCs were successfully transfected with LV6-TL1A and then stimulated with 10 μg/mL LPS for 6 h. The gene sequence ID was homo-*TL1A* (NM_005118.4).

Then, hPAEPICs were divided into the following groups to explore the effects of: (1) CTL group, with no interference, (2) LV6-DR3 group, in which hPAEPICs were only transfected with LV6-DR3, and cells successfully overexpressed DR3, (3) LPS group, stimulated by 10 μg/mL LPS for 6 h, and (4) LV6-DR3 + LPS, in which hPAEPCs were successfully transfected with LV6-DR3 and then stimulated with 10 μg/mL LPS for 6 h. The gene sequence ID was homo-*DR3* (NM_148965.2). hPAEPC transfection was performed following the GenePharma Recombinant Lentivirus Operation Manual.

Finally, hPAEPICs were divided into the CTL, Ctse, MMP-9, and HPA group to observe the effects of Ctse on the glycocalyx and tight junctions. hPAEPICs were cultured in 24-well plates until the density reached 70–80%. The recombinant human active Ctse (0.8 µg/mL), MMP-9 (0.8 µg/mL), and HPA (0.8 µg/mL) proteins were added for 6 h.

### Animals

The care and use of mice were performed strictly in accordance with the National Institutes of Health guidelines for the care and use of laboratory animals, and the study was approved by The First Affiliated Hospital of Shandong First Medical University and Shandong Provincial Qianfoshan Hospital Institutional Review Board.

C57BL/6 male mice (*n* = 8/group, 6–8 weeks old, 18–20 g) were bred in Jinan Pengyue Animal Breeding. The mice in the negative control group were intraperitoneally injected with PBS (200 μL). The mice in the LPS group were intraperitoneally injected with LPS (20 mg/kg, 200 μL) for 2, 4, and 6 h, and lung tissues were collected at different time points.

*TNFSF15 (TL1A)* KO mice with the C57BL/6 background were generated by CRISPR/Cas9 technology at the Model Animal Research Center of Nanjing University. Then, the mice in the CTL-TL1A^+/+^ or CTL-TL1A^−/−^ groups were intraperitoneally injected with PBS (200 μL, *n* = 8/group). The mice in the LPS + TL1A^+/+^ or LPS + TL1A^−/−^ groups were intraperitoneally injected with LPS (20 mg/kg, 200 μL, *n* = 8/group). The mice were stimulated for 6 h by LPS and injected with FITC-dextran (10 mg/kg, 200 μL) through the tail vein 1 h before euthanization, and then alveolar lavage fluid (BALF) and lung samples were collected [24].

TL1A alveolar epithelial CKO mice with the C57BL/6 background were generated by adeno-associated virus 6 (AAV6)-SP-C-TL1A technology from GeneChemCo (3.265E+11 v.g./mouse/tail vein injection). The gene sequence ID was TL1A-RNAi (111794-1). Then, the mice were intraperitoneally injected with PBS (200 μL) in the CTL-AT-II-NC shRNA or CTL-AT-II-TL1A shRNA groups (*n* = 8/group). The mice in the LPS-AT-II-NC shRNA or LPS-AT-II-TL1A shRNA groups were intraperitoneally injected with LPS (20 mg/kg, 200 μL, *n* = 8/group). The mice were treated with AAV-6 vectors for 30 days in vivo. After 6 h, the mice were intraperitoneally injected with LPS and then injected with FITC-dextran via the tail vein. BALF and lungs were collected.

*TNFRSF25 (DR3)* alveolar epithelial cell CKO mice with the C57BL/6 background were generated by CRISPR/Cas9 technology at Shanghai Model Organisms Center. DR3-CKO mice were rapidly bred with Sftpc-CreERT2 from the F0 generation, and floxed homozygous Cre-positive mice and the negative group were obtained by in vitro fertilization. Then, the mice were intraperitoneally injected with PBS (200 μL) in the CTL-DR3^flox/flox^ or CTL-CreERT2-DR3^flox/flox^ groups (*n* = 8/group). The mice in the LPS-DR3^flox/flox^ or LPS-CreERT2-DR3^flox/flox^ groups were intraperitoneally injected with LPS (20 mg/kg, 200 μL, *n* = 8/group). DR3^flox/flox^ and CreERT2-DR3^flox/flox^ mice were injected with FITC-dextran in the tail vein after LPS stimulation for 6 h, and lung samples were collected.

Ctse alveolar epithelial conditional OE mice with the C57BL/6 background were generated by AAV6-SP-C-Ctse technology from GeneChemCo (3.1E+11 v.g./mouse/tail vein injection). The target gene sequence was Ctse (NM_007799-P2A-LUC). The mice in the CTL-AT-II-NC or CTL-AT-II-Ctse OE groups were intraperitoneally injected with PBS (200 μL, *n* = 8/group). The mice in the LPS-AT-II-NC OE or LPS-AT-II-Ctse OE groups were intraperitoneally injected with LPS (20 mg/kg, 200 μL, *n* = 8/group). The mice were treated with AAV-6 OE vectors for 1 month, followed by LPS stimulation and the collection of BALF and lung tissue samples.

### Pathological staining

Lung tissues were fixed with 4% paraformaldehyde, dehydrated in ethanol, embedded in paraffin, and cut into 4 μm-thick sections. Pathological staining and evaluation were performed according to the previous study [25].

### Label-free quantitative proteomics

Approximately 500 µL of SDT lysate was added to different lung tissue samples. The samples were crushed by ultrasound treatment and homogenized. The supernatant was obtained by centrifugation at 16,000×*g* for 20 min, and protein quantification was performed using the BCA method. Then, 100 μg of protein from each lung tissue sample was digested by FASP, the digested peptide was dried and redissolved in 0.1% TFA, and the peptide concentration was determined for liquid chromatography–mass spectrometry (LC–MS) analysis. An appropriate amount of peptide was obtained from each sample for chromatographic separation by using the Easy nLC 1200 chromatography system (Thermo Scientific). The final LC–MS/MS RAW files were imported into the search engine Sequest HT in Proteome Discoverer software (Thermo Scientific) for database retrieval. The above work was completed by Shanghai Bioprofile Technology Company Ltd. (Shanghai, China).

### mRNA sequencing

Lung and hPAEPICs samples were enriched for mRNA with polyA structures by using Oligo(dT) magnetic beads. The RNA was broken into fragments with size of approximately 300 bp by ion interruption. RNA was used as template to synthesize first-strand cDNA by using six-base random primers and reverse transcriptase, and the first-strand cDNA was used as template for second-strand cDNA synthesis. PCR amplification was used to enrich the library fragments after library construction, followed by size selection. The library size was 450 bp. Then, the library was evaluated using the Agilent 2100 Bioanalyzer to detect the total and effective concentration. The libraries containing different index sequences were mixed proportionally according to the effective concentration of the library and the amount of data required. The pooled libraries were uniformly diluted to 2 nM, and single-stranded libraries were formed by alkali denaturation. Paired-end sequencing was performed using the Illumina HiSeq sequencing platform after RNA extraction, purification, and library construction. Sequencing analysis was carried out by Shanghai Bioprofile Technology Company Ltd. (Shanghai, China).

### Single cell RNA-sequencing

The single cell suspensions in lung tissue samples were prepared, and their quality was inspected to ensure cell activity. Individual cells, reagents required for the reaction, and gel beads with the cell barcode were wrapped in oil droplets to generate gel beads in emulsion (GEMs). Cell lysis released RNA in the GEMs. Under appropriate conditions, RNA was combined with Poly(dT) primers with the cell barcode and UMI to extend the complementary strand, and three C bases were added to the end of the extended strand. TSO was used for template extension to complete the reverse transcription reaction after the complementary pairing of CCC and rGrGrG of TSO. Subsequently, GEMs were broken, and cDNA was recovered and enriched by PCR amplification for library construction. Qubit 4.0 was used to determine the cDNA concentration and Agilent 2100 was used to evaluate cDNA integrity. A second-generation sequencing platform was used for sequencing after quality control. Single-cell RNA sequencing and bioinformatics analyses were performed by Biomarker Technologies Corporation (http://www.biocloud.net).

### Mouse AT II cell isolation and culture

TL1A CKO mice were sufficiently anesthetized with isoflurane, and the mice (except their heads) were promptly disinfected with 75% alcohol. The trachea and ventral aorta were quickly separated on an ice brick of a superpurgative working table. The lung was removed and placed in pre-cooled PBS to remove the trachea. It was cut into sections with dimension of 1–5 mm^3^ and transferred to a 15 mL sterile tube. An appropriate amount of 0.25% trypsin and type I collagenase were added at a 1:1 ratio. The tube was placed in a water bath at 37 °C for 20 min. Dulbecco’s modified Eagle’s medium (DMEM) was added to stop digestion, and an appropriate amount of DNase I was added to the water bath for 5 min at 37 °C. The digested cells were filtered through a 200-mesh cell screen and centrifuged at 500×*g* and 4 °C for 8 min, and the supernatant was discarded. Then, cells were resuspended in red blood cell lysis buffer and centrifuged at 500×*g* and 4 °C for 8 min before the supernatant was discarded. The cells were resuspended in DMEM and added to a six-well plate containing mouse IgG. Then, the cells were placed in an incubator with 5% CO_2_ and 37 °C for 1 h. Non-adherent cells were sucked out and centrifuged at 500×*g* at 4 °C for 8 min. Alveolar type II (AT II) cells were counted and inoculated into the six-well plate to observe the growth of adherent cells. Mouse AT II cell isolation and culture were performed as described by Chen et al. [26].

### Immunohistochemistry

Lung tissue sections were treated with 3% H_2_O_2_ to remove endogenous peroxidase and repaired with 0.01 M sodium citrate buffer (pH 6.0). The goat serum was blocked at 37 °C for 20 min, and the excess liquid was shaken off. The TL1A and DR3 antibodies were then incubated at 4 °C. After the next day, the lung tissue sections were washed with PBS solution (three times, 6 min each), and the Bio-sheep anti-rabbit IgG working solution was dropped and incubated at room temperature for 30 min. After washing with PBS, the streptavidin-peroxidase working solution was added dropwise and incubated for 30 min at room temperature, and the sections were stained with DAB and hematoxylin. Finally, the sections were dehydrated until transparent, sealed, and observed under a microscope. ImageJ was used to assess the protein expression of immunohistochemistry staining (IHC).

### Western blot

The protein samples were subjected to 10% SDS polyacrylamide gel electrophoresis and then transferred to a PVDF membrane at a low temperature. The PVDF membrane was washed by TBST (three times, 5 min each) and incubated with 5% BSA for 60 min. The PVDF membrane was washed by TBST (three times, 5 min each), then incubated with antibodies (TL1A, DR3, Ctse, SDC-1, Tjp-3, and GAPDH) at 4 °C for at least 12 h. The PVDF membrane was washed with TBST (three times, 10 min each), and incubated with goat anti-rabbit IgG HRP for 60 min. Then, the PVDF membrane was washed with TBST (three times, 15 min each), and the PVDF membrane was developed.

### Immunofluorescence

Lung tissues were thermally repaired with 0.01 M sodium citrate buffer (pH 6.0) antigen after dewaxing and dehydration. After the tissue sections reached room temperature, they were washed with PBS (three times, 5 min each) and incubated with TL1A, DR3, Ctse, Tjp-3, SDC-1, and SP-C antibodies at 4 °C for 12 h. Sections were raised to room temperature and washed with PBS (three times, 5 min each) before staining with Alexa Fluor 488 or 549 and DAPI. Finally, the sections were washed with PBS (three times, 10 min each). The samples were imaged under a fluorescence microscope (Olympus, Tokyo, Japan).

hPAEPICs or AT-II cells were inoculated on a 24-well plate. The cells were fixed with 4% paraformaldehyde after different treatments, washed with PBS (three times, 5 min each), and incubated with TL1A, FITC-dextran, Ctse, Tjp-3, and SDC-1 antibodies at 4 °C for 12 h. hPAEPICs or mouse primary AT II cells were washed with PBS (three times, 5 min each) and stained with Alexa Fluor 488 or 549 and DAPI. Finally, the cells were observed and imaged under a fluorescence microscope (Olympus) after washing with PBS (three times, 5 min each).

Fluorescence intensity is a semi-quantitative analysis. The relative quantitative density was performed by Image J software. The relative quantitative intensity = total fluorescence intensity of the area / area or number of cells in the area.

### Measurement of FITC-dextran in BALF

The neck and main bronchus were progressively exposed after mice were anesthetized with isoflurane. The trachea was inserted into the trocar, and the needle core was removed. Then, a 1 mL syringe with 0.6 mL of sterile PBS was connected to the other end of the trocar. BALF was obtained while avoiding light with a pressure difference. BALF was added to a 96-well plate, and the permeability of mouse alveolar epithelial cells was evaluated by fluorescent enzyme labeling at an excitation wavelength of 490 nm and emission wavelength of 520 nm [27].

### Transwell assay

The upper chamber of a 12-well Transwell plate was filled with hPAEPICs in an incubator with 5% CO_2_ and 37 °C. FITC-dextran (0.5 mg/mL, 100 μL) was added to the upper chamber after different treatments and then incubated for 60 min. Then, 100 μL of the lower chamber solution was added to a 96-well culture plate, and the fluorescence intensity was detected under an excitation wavelength of 490 nm and emission wavelength of 520 nm [27]. The permeability of alveolar epithelial cells in vitro was measured by measuring the fluorescence of the culture medium in the lower chamber.

### Electron microscopy

The mice were anesthetized with isoflurane, and the chest cavity was progressively opened to expose the heart and lung tissue. The right ventricle was perfused with ixative solution (2% glutaraldehyde, 2% sucrose, 0.1 M sodium diarsenate buffer, and 2% lanthanum nitrate). The lung tissue was removed, cut into sections with dimensions of 2 mm × 2 mm × 2 mm, rinsed, and fixed with difloxacin sodium buffer. Lung tissue blocks were embedded in 2% osmium tetroxide and 2% lanthanum nitrate solution, and sections were prepared for electron microscopy. Electron microscopy was performed as described by Inagawa et al. [28].

### Statistical analysis

Data are expressed as the mean ± standard deviation. Differences between two groups were analyzed by* t-*test, and differences among more than two groups were analyzed by one-way ANOVA. Data analyses were performed using GraphPad Prism 9.4.1. Values of p < 0.05 were considered statistically significant.

## Results

### DR3 levels are decreased in the alveolar epithelium in ARDS

**Fig. 1:**
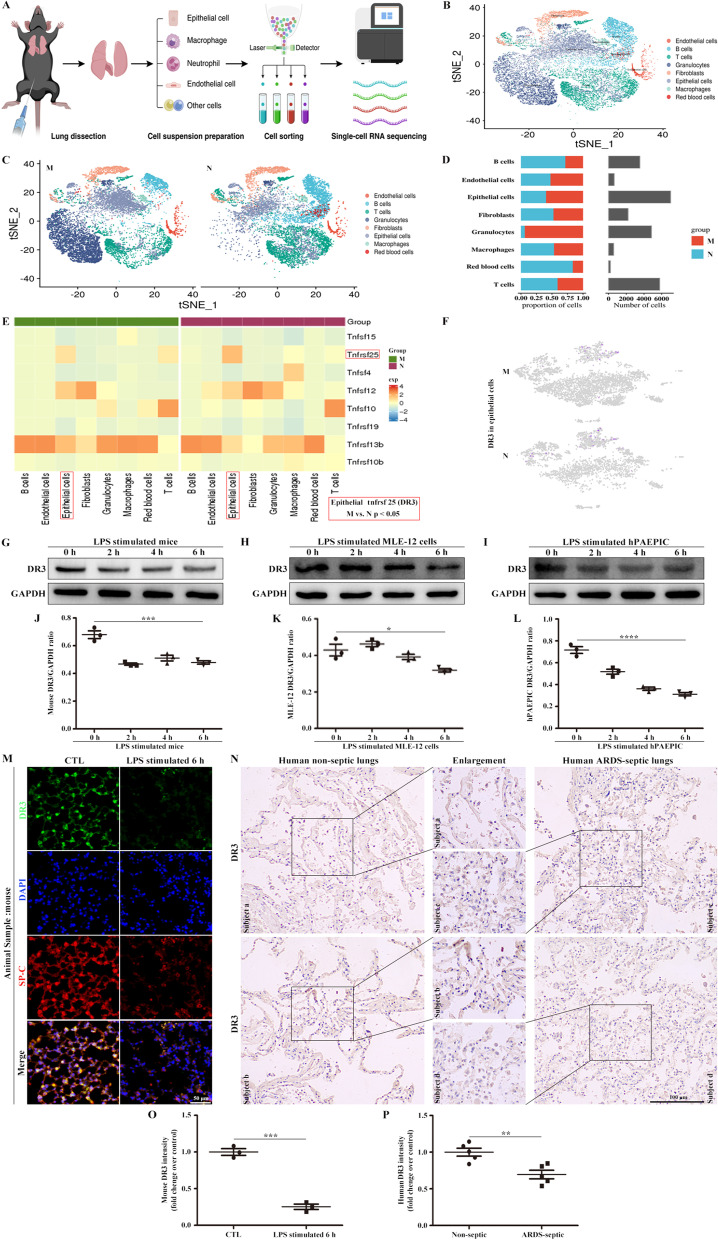
DR3 expression decreased in the alveolar epithelium in ARDS. **A** Schematic single-cell RNA sequencing of lung tissues isolated from mice treated with PBS (N) or LPS (M). **B** The t-distributed stochastic neighbor embedding (t-SNE) plot with all tissues. **C** The t-SNE plot with N and M groups. **D** Histogram represents changes in cell counts under PBS or LPS treatment. **E** Differential gene expression of some TNF family members in different cells; note that the DR3 expression is significantly differentially expressed in epithelial cells (red bracket). **F** Distribution of *DR3* gene expression in epithelial cells after PBS or LPS treatment. **G** LPS-stimulated mice, **H** LPS-stimulated MLE-12 cells, and **I** LPS-stimulated hPAEPICs were evaluated for the expression of DR3 by Western blot. **J** DR3/GAPDH ratio of mice. **K** DR3/GAPDH ratio of MLE-12 cells. **L** DR3/GAPDH ratio of hPAEPICs. **M** Immunofluorescence staining of DR3 from mouse lung sections. DR3 (green), SP-C (red), and DAPI (blue). Scale bar, 50 μm. **N** IHC staining of DR3 from human lung sections. Scale bar, 100 μm. **O** DR3 fluorescence intensity analysis of mice. **P** DR3 intensity analysis of human samples. Data are expressed as the means ± SD of three independent experiments. *p < 0.05, **p < 0.01, ***p < 0.001, ****p < 0.0001. *NS* not significant

Lung samples from mice were subjected to single-cell RNA sequencing to evaluate the differences between normal mice and mice with LPS-induced ARDS (Fig. 1A). The mice were treated with LPS for 6 h, resulting in severe lung injury and dramatic changes in cell types. A total of 11,025 and 13,762 cells from control and LPS-induced ARDS mice were analyzed. As determined by the expression of lineage-specific markers, the main cell types were endothelial cells, B cells, T cells, neutrophils, fibroblasts, epithelial cells, and macrophages (Fig. 1B–D). Gene enrichment analysis was performed to observe changes in the receptor *DR3* in single cells of ARDS mice. DR3 levels differed among cells, but the distribution of *DR3* in epithelial cells in ARDS mice was significantly lower than that in the normal group (Fig. 1E, F). Further analyses were performed to verify these findings. Combined with the LPS-stimulated cell activity (Additional file 1) and the previous articles [22, 23], LPS-stimulated concentration was selected as 10 μg/mL. Western blot analysis indicated that DR3 levels in mice, MLE-12 cells, and hPAEPICs decreased significantly after LPS stimulation for 6 h (Fig. 1G–L). The colocalization of DR3 and SP-C remarkably decreased after LPS stimulation compared with the normal group, as determined by alveolar epithelial cell marker detection (Fig. 1M, O). In addition, DR3 levels in the human ARDS-septic alveolar epithelium were substantially lower than those in control samples as determined by IHC (Fig. 1N, P). Therefore, DR3 in the alveolar epithelium in ARDS remarkably decreased.

### TL1A in the alveolar epithelium is significantly reduced in ARDS

**Fig. 2:**
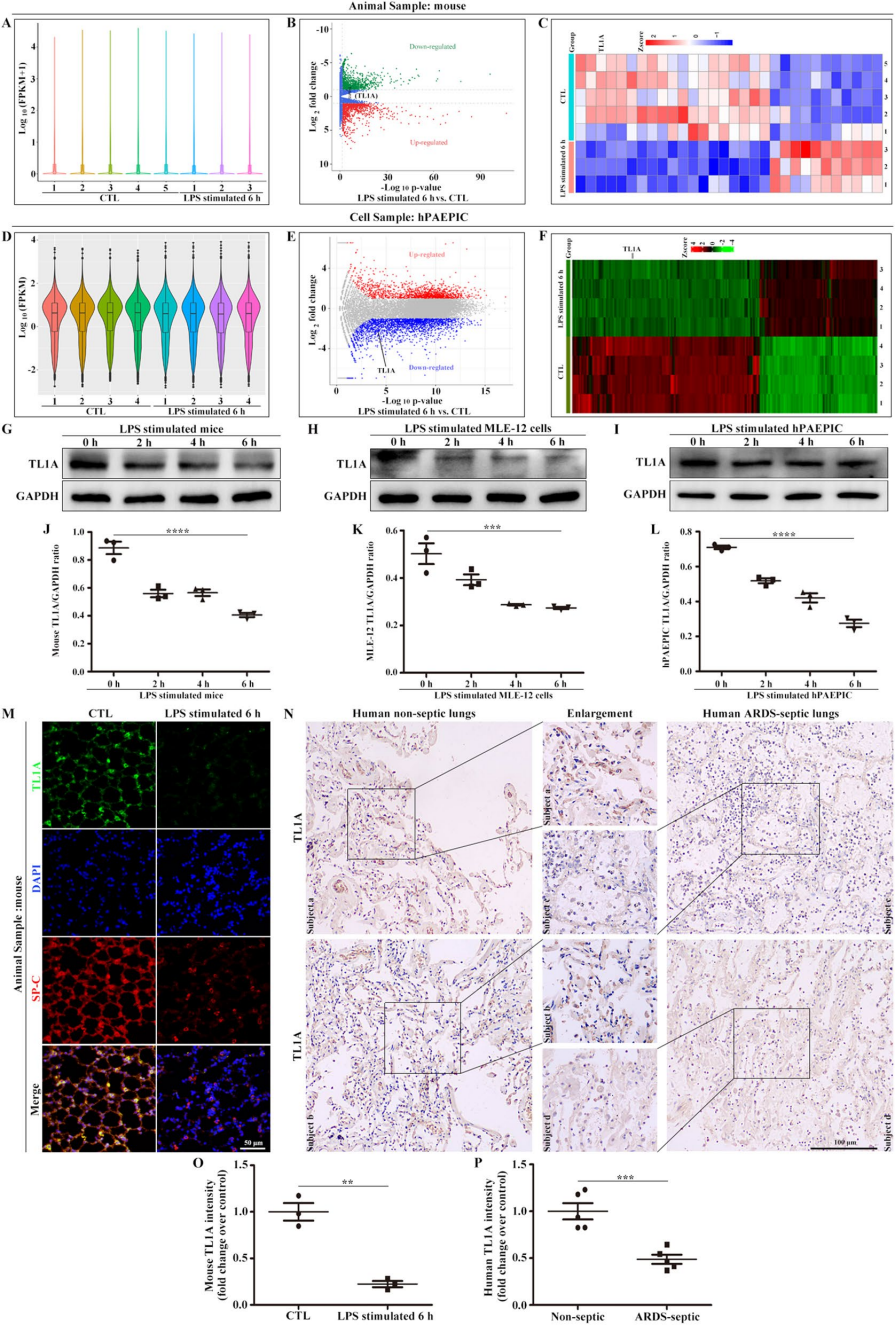
TL1A expression decreased in the alveolar epithelium in ARDS. **A** A violin map representing data quality and repeatability in mouse samples. **B** The criteria for the identification of differentially expressed proteins (DEPs) are p < 0.05 and fold change > 1.5 in volcano plots. **C** Heatmap maps showing the distribution of DEPs. **D** A violin map representing the data quality and repeatability in hPAEPIC samples. **E** The criteria for the identification of DEPs are p < 0.05 and fold change > 1.5 in volcano plots. **F** Heatmap maps showing the distribution of DEPs. **G** TL1A changes in LPS-stimulated mice. **H** TL1A changes in LPS-stimulated MLE-12 cells. **I** TL1A changes in LPS-stimulated hPAEPICs. **J** Mouse TL1A/GAPDH ratio. **K** TL1A/GAPDH ratio of LPS-stimulated MLE-12 cells. **L** TL1A/GAPDH ratio of LPS-stimulated hPAEPICs. **M** Immunofluorescence staining of TL1A from mouse lung sections. TL1A (green), SP-C (red), and DAPI (blue). Scale bar, 50 μm. **N** IHC staining of TL1A from human lung sections. Scale bar, 100 μm. **O** TL1A fluorescence intensity analysis of mice. **P** TL1A intensity analysis of human samples. Data are expressed as the means ± SD of three independent experiments. *p < 0.05, **p < 0.01, ***p < 0.001, ****p < 0.0001. NS, not significant

Changes in TL1A after LPS stimulation in mice and hPAEPICs were further identified via RNA transcriptome analysis. Points in a violin map were close to mean value, indicating good data quality and repeatability in mouse and hPAEPICs samples (Fig. 2A, D). Volcano plots and heatmaps showed that TL1A was remarkably lower in LPS-stimulated mice and hPAEPICs than in the corresponding normal groups (Fig. 2B, C, E, F). Next, Western blot analysis results showed that the expression of TL1A, the ligand of DR3, was substantially reduced in mice or MLE-12 or hPAEPICs after LPS stimulation for 6 h (Fig. 2G–L). Immunofluorescence based on the co-localization of TL1A and SP-C showed that TL1A was notably reduced in the alveolar epithelium of mice stimulated by LPS compared with that in the normal group (Fig. 2M, O). In addition, alveolar epithelial TL1A was remarkably lower in patients with ARDS-related sepsis than in healthy individuals, as determined by IHC (Fig. 2N, P).

The reduced expression levels of TL1A and DR3 further indicate that the TL1A/DR3 axis plays an important role in the pathogenesis of ARDS.

### TL1A KO aggravated pulmonary edema and inflammation with LPS-induced ARDS mice

**Fig. 3:**
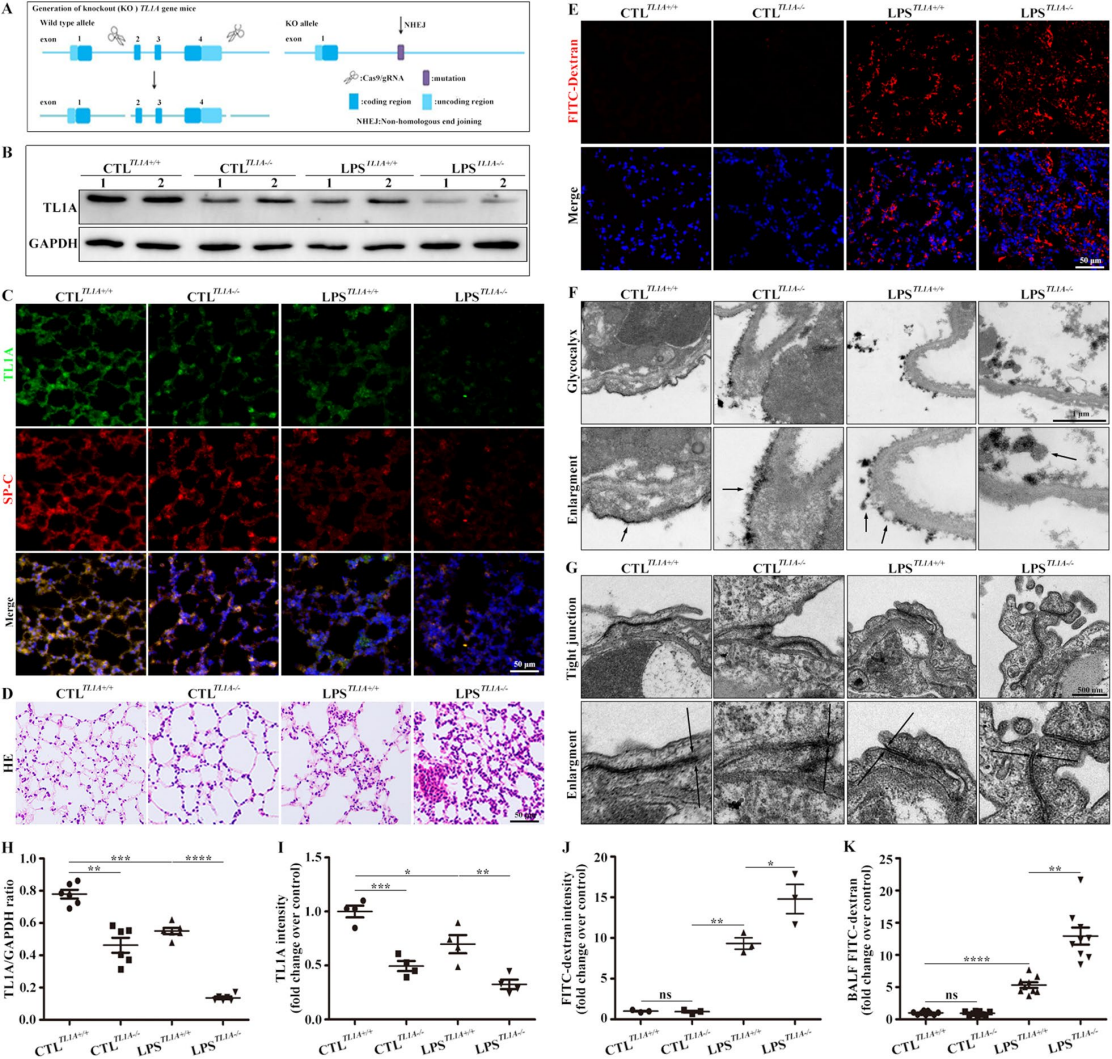
TL1A KO promoted inflammatory responses and pulmonary edema in LPS-induced ARDS. **A** Strategy for TL1A KO. **B** Changes in TL1A levels were verified by Western blot analysis. **C** Immunofluorescence staining of TL1A from mouse lung sections. TL1A (green), SP-C (red), and DAPI (blue). Scale bar, 50 μm. **D** Pathological damage was observed by HE. Scale bar, 50 μm. **E** Immunofluorescence staining of FITC-dextran (red), and DAPI (blue). Scale bar, 50 μm. **F** Glycocalyx microstructures observed by electron microscopy. Scale bars, 1 μm. **G** Tight junction microstructures observed by electron microscopy. Scale bars, 500 nm. **H** Mouse TL1A/GAPDH ratio. **I** TL1A intensity analysis of lungs. **J** FITC-dextran fluorescence intensity analysis of lungs. **K** Intensity of FITC-dextran in the BALF. Data are expressed as the means ± SD of three independent experiments. *p < 0.05, **p < 0.01, ***p < 0.001, ****p < 0.0001. *NS* not significant

TL1A KO mice were generated to further explore the role of TL1A in ARDS (Fig. 3A). TL1A protein levels were significantly lower in TL1A KO mice than in wild-type (WT) mice, confirming the successful construction of TL1A KO mice (Fig. 3B, C, H, I). LPS-stimulated TL1A KO mice showed more severe pathological damage in lung tissues than that in LPS-stimulated WT mice (Fig. 3D). The localization of FITC-dextran immunofluorescence staining and the intensity of FITC-dextran in BALF confirmed that TL1A affected lung permeability in ARDS mice (Fig. 3E, J, K). Lung permeability is closely related to the glycocalyx and tight junctions. Therefore, microscopic changes in the glycocalyx on the cell surface and tight junctions between cells were examined by electron microscopy. The loss of TL1A resulted in increased glycocalyx shedding and the relaxation of tight junctions in lung tissues of ARDS mice (Fig. 3F, G).

### TL1A CKO aggravated pulmonary edema by damaging alveolar epithelium in LPS-induced ARDS mice

**Fig. 4:**
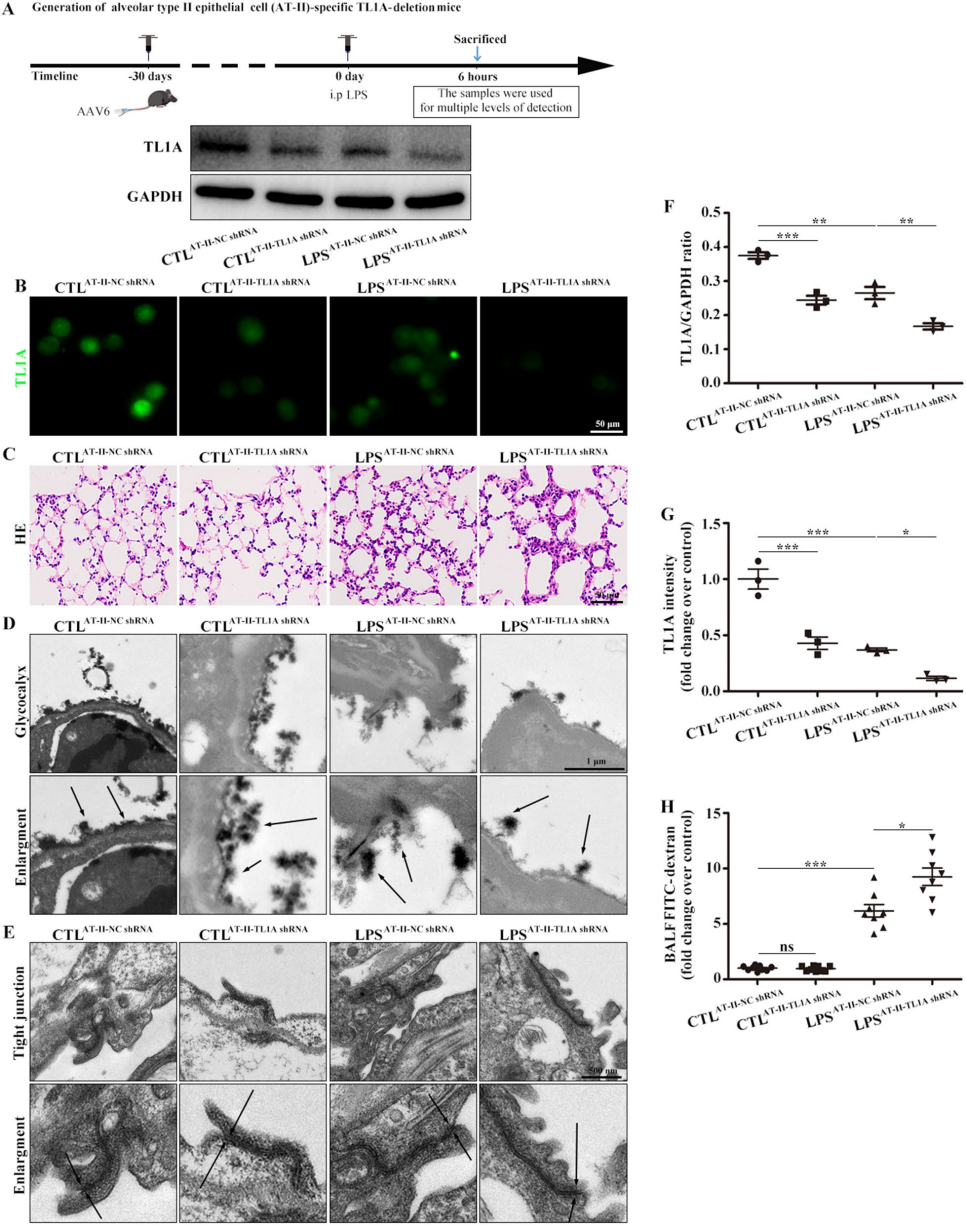
TL1A CKO promoted inflammatory infiltration and pulmonary edema by damaging the alveolar epithelial barrier. **A** Strategy for the establishment of TL1A CKO. Changes in TL1A levels were verified by Western blot analysis. **B** Immunofluorescence staining of TL1A (green) in primary alveolar epithelial cells from mice. Scale bar, 50 μm. **C** Pathological damage was observed by HE. Scale bar, 50 μm. **D** Glycocalyx microstructures observed by electron microscopy. Scale bars, 1 μm. **E** Tight junction microstructures observed by electron microscopy. Scale bars, 500 nm. **F** Mouse TL1A/GAPDH ratio. **G** TL1A fluorescence intensity analysis of mouse cells. **H** Intensity of FITC-dextran in BALF. Data are expressed as the means ± SD of three independent experiments. *p < 0.05, **p < 0.01, ***p < 0.001, ****p < 0.0001. *NS* not significant

TL1A CKO mice were generated by injecting AAV6-TL1A shRNA through the caudal vein to further explore the role of TL1A in ARDS (Fig. 4A, F). AT-II cells isolated from TL1A CKO mice showed a significant reduction in TL1A expression (Fig. 4B, G). HE staining results confirmed that TL1A CKO aggravated the ARDS disease pathology (Fig. 4C). Furthermore, TL1A CKO exacerbated the glycocalyx shedding and the relaxation of tight junctions by electron microscopy (Fig. 4D, E). FITC-dextran intensity analyses in BALF confirmed that TL1A CKO plays a key role in lung permeability (Fig. 4H). Therefore, TL1A plays a protective role in lung permeability.

### TL1A deficiency promoted Ctse release and decreased SDC-1 and Tjp-3 expression in LPS-induced TL1A KO mice

**Fig. 5:**
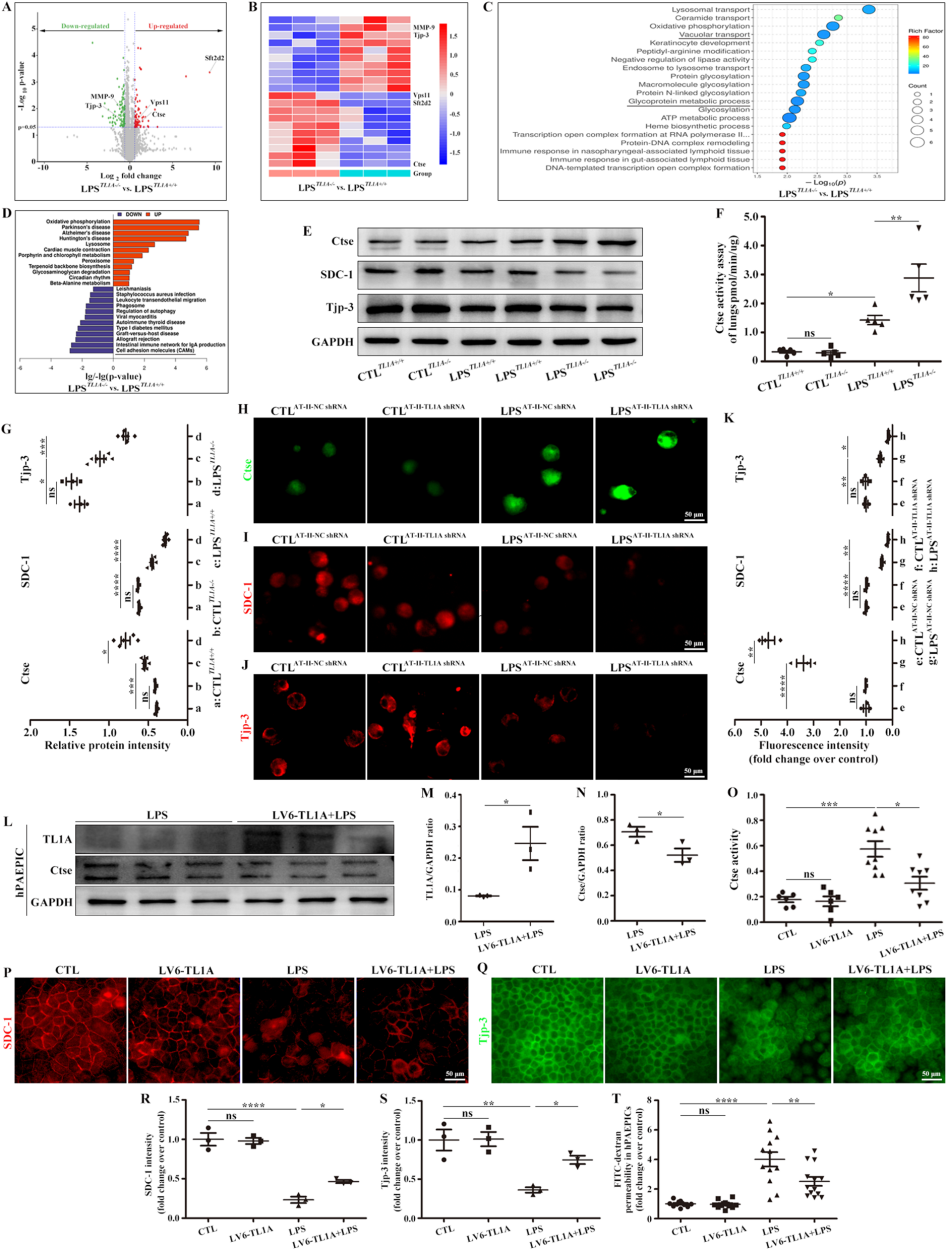
TL1A deficiency (KO and CKO) decreased the levels of SDC-1 and Tjp-3 and increased the expression and activity of Ctse. **A** Volcano plots for screening DEPs with thresholds of p < 0.05 and fold change > 1.5. **B** Gene heatmaps show upregulated and downregulated genes. **C** The top 20 significantly enriched function after TL1A KO. **D** The top 10 disease associations enriched after TL1A KO. **E** Changes in Ctse, SDC-1, and Tjp-3 were verified by Western blot analysis. **F** Ctse activity in lungs was observed by ELISA. **G** Protein intensity (Ctse, SDC-1, and Tjp-3) analysis of LPS-stimulated mice. Immunofluorescence staining of **H** Ctse, **I** SDC-1, and **J** Tjp-3 in primary alveolar epithelial cells from mice. Ctse (green), SDC-1 (red), and Tjp-3 (red). Scale bar, 50 μm. **K** Fluorescence intensity analysis of the primary alveolar epithelial cells from mice. **L** Changes in TL1A and Ctse in hPAEPICs as determined by Western blot analysis. **M** hPAEPIC TL1A/GAPDH ratio. **N** hPAEPIC Ctse/GAPDH ratio. **O** Ctse activity in hPAEPICs was observed by ELISA. Effect of TL1A OE on LPS-induced **P** SDC-1 and **Q** Tjp-3 as demonstrated by immunofluorescence. SDC-1 (red) and Tjp-3 (green). Scale bars, 50 μm. **R** SDC-1 fluorescence intensity analysis of hPAEPICs. **S** Tjp-3 fluorescence intensity analysis of hPAEPICs. **T** FITC-dextran in the Transwell assay was used to assess to cell permeability. Data are expressed as the means ± SD of three independent experiments. *p < 0.05, **p < 0.01, ***p < 0.001, ****p < 0.0001. *NS* not significant

The criteria for screening DEPs were p < 0.05 and fold change > 1.5 (Fig. 5A). Volcano plots and gene heatmaps revealed various upregulated genes (*Vps11, Ctse*, and *Sft2d2*) and downregulated genes (*MMP-9* and *Tjp-3*, Fig. 5A, B). The top 20 significant DEPs between groups included proteins related to vacuolar transport and glycoprotein metabolic processes (Fig. 5C). Figure 5D shows the top 10 proteins enriched for disease association after TL1A KO, revealing a decrease in cell adhesion molecules. This finding indicates that TL1A KO may affect the glycoproteins SDC-1 and Tjp-3 via Ctse, thus affecting permeability. Next, Western blot analysis results confirmed that TL1A KO increased Ctse expression and activity but decreased SDC-1 and Tjp-3 in ARDS (Fig. 5E–G).

### TL1A CKO also promoted Ctse release and decreased SDC-1 and Tjp-3 expression

Primary mouse AT-II cells were further evaluated by immunofluorescence, and the results show that the specific deletion of TL1A increased Ctse expression and decreased SDC-1 and Tjp-3 levels (Fig. 5H–K). In addition, TL1A-overexpressing hPAEPICs were constructed. TL1A OE remarkably saved the LPS-induced reduction of TL1A and then inhibited LPS-induced Ctse expression and activity (Fig. 5L–O). TL1A OE also remarkably increased the expression of SDC-1 and Tjp-3 (Fig. 5P–S) and improved LPS-induced cell permeability (Fig. 5T).

### Ctse decreased SDC-1 and Tjp-3 expression in the alveolar epithelium

**Fig. 6:**
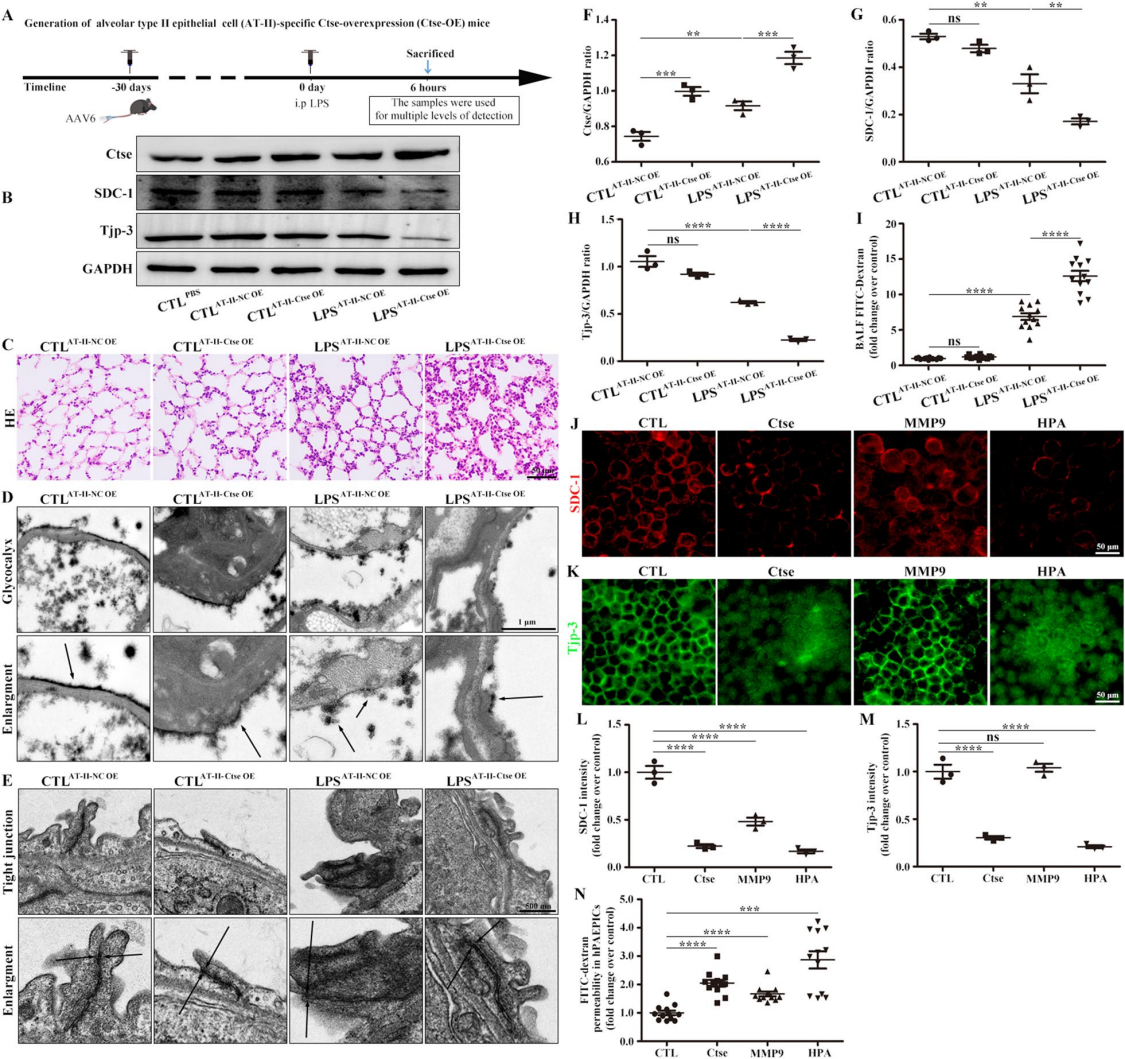
Ctse OE in the mouse alveolar epithelium decreased the levels of SDC-1 and Tjp-3 in LPS-induced ARDS and active human recombinant Ctse protein decreased SDC-1 and Tjp-3 in hPAEPICs. **A** AT-II-overexpressing cells in mouse lungs were constructed. **B** Changes of Ctse, SDC-1, and Tjp-3 were detected by Western blot analysis. **C** Pathological damage was observed by HE. Scale bar, 50 μm. **D** Glycocalyx microstructures observed by electron microscopy. Scale bars, 1 μm. **E** Tight junction microstructures observed by electron microscopy. Scale bars, 500 nm. **F**, **G**, and **H** Protein intensity analysis of Ctse, SDC-1, and Tjp-3. **I** Intensity of FITC-dextran in BALF. Effects of active human recombinant proteins Ctse, MMP-9, and HPA on hPAEPICs. **J** SDC-1 and **K** Tjp-3 were detected by immunofluorescence. SDC-1 (red) and Tjp-3 (green). Scale bars, 50 μm. **L**, **M** SDC-1 and Tjp-3 fluorescence intensity analysis. **N** FITC-dextran in the Transwell assay was used to assess the cell permeability. Data are expressed as the means ± SD of three independent experiments. *p < 0.05, **p < 0.01, ***p < 0.001, ****p < 0.0001. *NS* not significant

AT-II cells overexpressing Ctse were used to further explore the role of Ctse in ARDS (Fig. 6A). Western blot analysis results confirmed that the model of Ctse OE in the epithelium was successfully established (Fig. 6B, F), and the protein levels of SDC-1 and Tjp-3 in the LPS-AT-II Ctse-OE group were remarkably lower than those in the LPS-AT-II NC-OE group (Fig. 6B, G, H). Pathological damage, edema, and inflammatory cells were higher in the LPS-AT-II Ctse-OE group than in the LPS-AT-II NC-OE group based on HE staining and BALF FITC-dextran (Fig. 6C, I). Electron microscopy results revealed that the damage to tight junctions and the glycocalyx structure in the LPS-AT-II Ctse-OE group was more obvious than that in the LPS-AT-II NC-OE group (Fig. 6D, E). Finally, treatment with active human recombinant proteins Ctse, MMP-9, and HPA revealed that active human recombinant Ctse significantly decreased SDC-1 and Tjp-3 levels in hPAEPICs (Fig. 6J–M), further increasing hPAEPICs permeability (Fig. 6N).

### DR3, the receptor for TL1A, is also required for the alveolar epithelial barrier

**Fig. 7:**
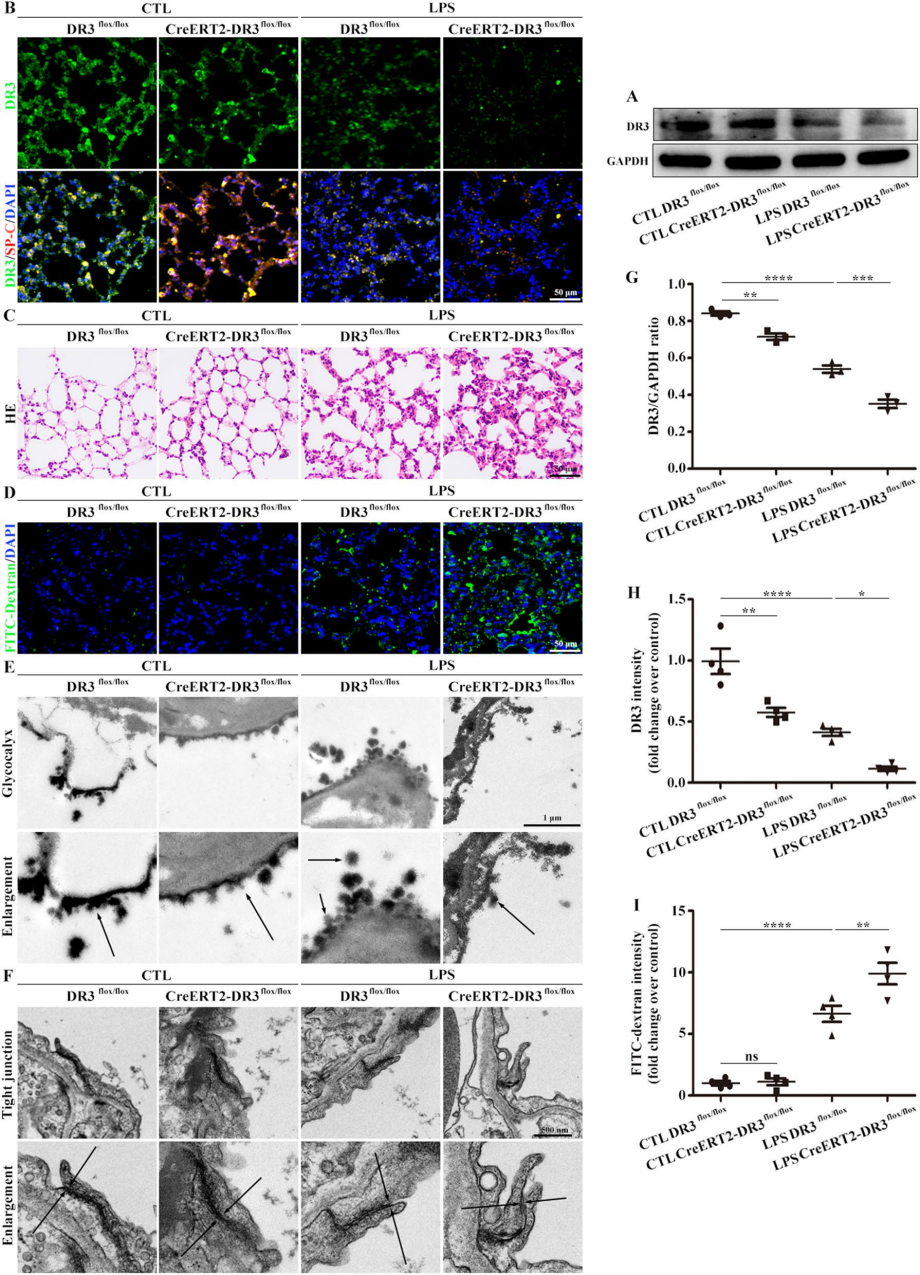
DR3 deficiency aggravated the inflammation and structures of the epithelial glycocalyx and tight junctions. **A** Changes of DR3 were detected by Western blot analysis. **B** Immunofluorescence staining of DR3 from DR3 CKO. DR3 (green), SP-C (red), and DAPI (blue). Scale bar, 50 μm. **C** HE was used to evaluate pathological damage. Scale bar, 50 μm. **D** Immunofluorescence staining of FITC-dextran from DR3 CKO. FITC-dextran (green) and DAPI (blue). Scale bar, 50 μm. **E** Glycocalyx microstructures observed by electron microscopy. Scale bars, 1 μm. **F** Tight junction microstructures observed by electron microscopy. Scale bars, 500 nm. **G** Mouse DR3/GAPDH ratio. **H** DR3 fluorescence intensity analysis. **I** FITC-dextran fluorescence intensity analysis. Data are expressed as the means ± SD of three independent experiments. *p < 0.05, **p < 0.01, ***p < 0.001, ****p < 0.0001. *NS* not significant

The experimental results indicate that TL1A, the ligand of DR3 in alveolar epithelial cells, plays a protective role in the development of ARDS. More importantly, its receptor DR3 was significantly reduced during the disease process. Accordingly, DR3-CKO mice was constructed. The expression of DR3 in DR3-CKO mice was remarkably reduced, as determined by Western blot analysis and immunofluorescence co-localization (Fig. 7A, B, G, H). Further evaluation revealed that DR3-CKO aggravated inflammation and pulmonary edema in LPS-induced ARDS (Fig. 7C, D, I). Electron microscopy results confirmed that DR3-CKO aggravated the microstructural changes of glycocalyx shedding and relaxation of tight junctions, which are closely related to permeability, in LPS-induced ARDS (Fig. 7E, F).

**Fig. 8:**
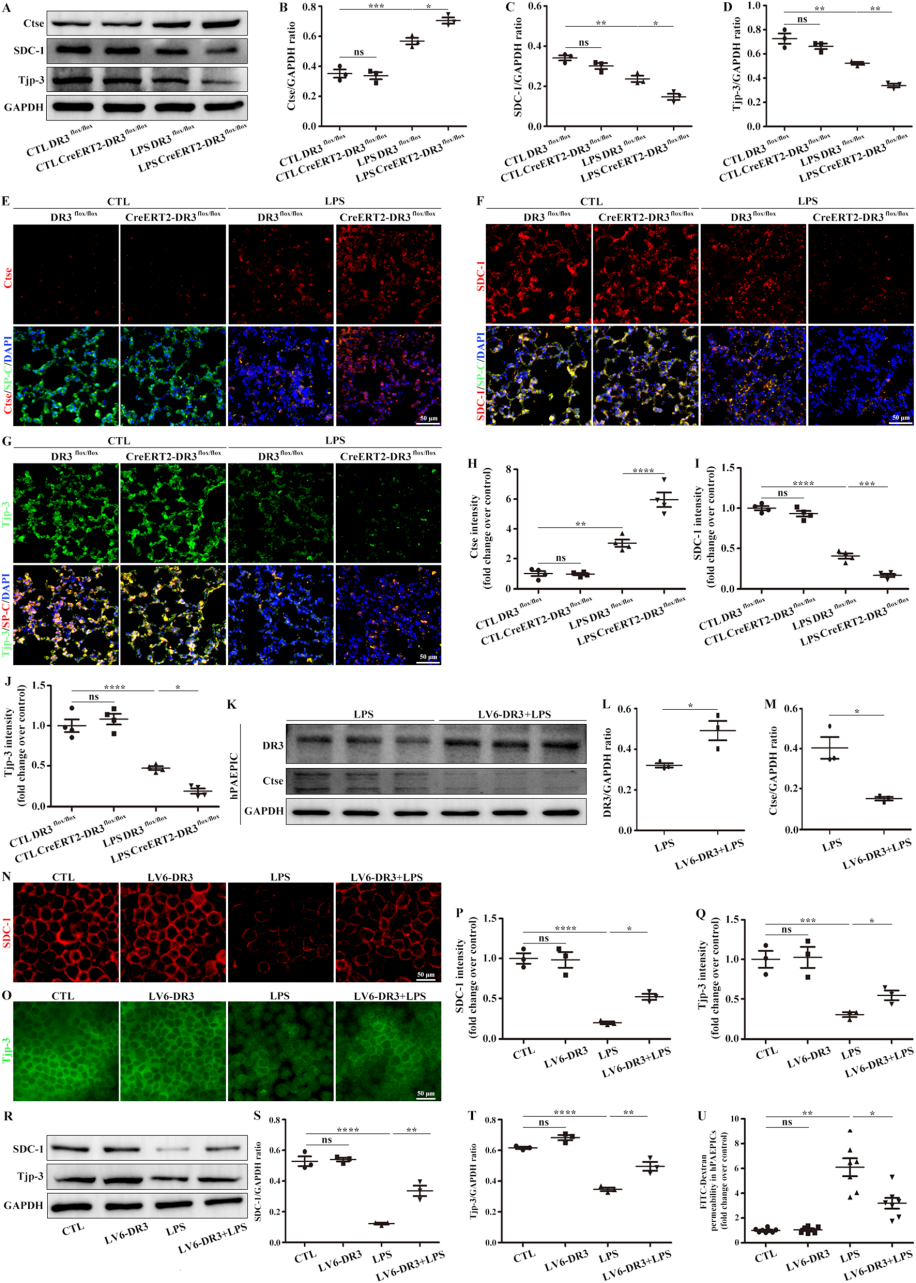
DR3 deficiency decreased the levels of SDC-1 and Tjp-3 by increasing the expression of Ctse. **A** Changes in Ctse, SDC-1, and Tjp-3 were detected by Western blot analysis. **B** Mouse Ctse /GAPDH ratio. **C** Mouse SDC-1/GAPDH ratio. **D** Mouse Tjp-3/GAPDH ratio. **E** Immunofluorescence staining for the detection of Ctse in DR3 CKO lung sections. Ctse (red), SP-C (green), and DAPI (blue). Scale bar, 50 μm. **F** Immunofluorescence staining for the detection of SDC-1 in DR3 CKO lung sections. SDC-1 (red), SP-C (green), and DAPI (blue). Scale bar, 50 μm. **G** Immunofluorescence staining for the detection of Tjp-3 in DR3 CKO lung sections. Tjp-3 (green), SP-C (red), and DAPI (blue). Scale bar, 50 μm. **H** Ctse, **I** SDC-1, and **J** Tjp-3 fluorescence intensity analysis of lungs. **K** Effects of DR3 OE in hPAEPICs on LPS-induced Ctse were detected by Western blot analysis. **L** DR3/GAPDH ratio in hPAEPICs. **M** Ctse/GAPDH ratio in hPAEPICs. Effects of DR3 OE in hPAEPICs on LPS-induced **N** SDC-1 and **O** Tjp-3 observed by immunofluorescence. SDC-1 (red), Tjp-3 (green). Scale bars, 50 μm. **P** SDC-1 and **Q** Tjp-3 fluorescence intensity analysis of hPAEPICs. **R** The effects of DR3 OE in hPAEPICs on LPS-induced SDC-1 and Tjp-3 were detected by Western blot analysis. **S** SDC-1/GAPDH ratio in hPAEPICs. **T** Tjp-3/GAPDH ratio in hPAEPICs. **U** FITC-dextran in the Transwell assay was used to assess the cell permeability. Data are expressed as the means ± SD of three independent experiments. *p < 0.05, **p < 0.01, ***p < 0.001, ****p < 0.0001. *NS* not significant

Based on the above findings, the glycocalyx component SDC-1 and tight junction component Tjp-3 were remarkably decreased in the alveolar epithelium, while the degradation enzyme Ctse was increased based on Western blot analysis and immunocolocalization in LPS-induced DR3-CKO mice compared with LPS-induced WT mice (Fig. 8A–J). DR3-overexpressing hPAEPICs were constructed for further experiments. DR3-overexpressing significantly inhibited the degradation enzyme Ctse (Fig. 8K–M), increased the levels of SDC-1 and Tjp-3 (Fig. 8N–T), and improved the permeability of epithelial cells (Fig. 8U).

## Discussion

**Fig. 9:**
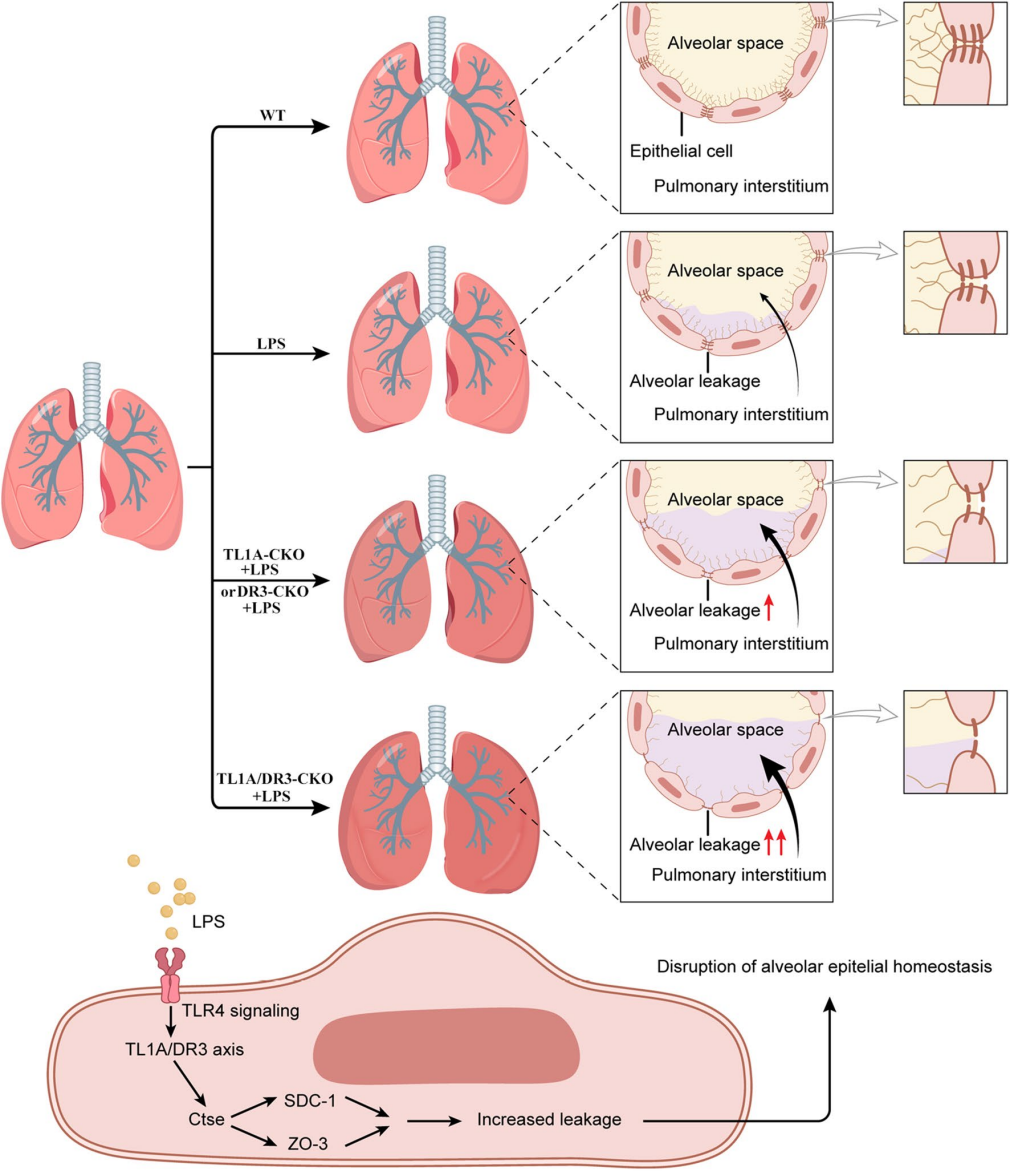
Scheme showing that a deficiency in the TL1A/DR3 axis exerts negative effects on SDC-1 and Tjp-3 by increasing Ctse signaling in the alveolar epithelium

Alveolar epithelial barrier dysfunction is among the main causes of pulmonary edema. This study provides the first demonstration that DR3 and its only known ligand TL1A have critical roles in the protection of alveolar epithelial barrier function. The TL1A/DR3 axis protects the integrity of the glycocalyx and tight junctions in the alveolar epithelium by inhibiting Ctse levels or activity (Fig. 9).

TL1A exists in soluble and membrane-bound forms, and it exerts effects by binding to DR3. TL1A is abundantly expressed in umbilical vein endothelial cells, T cells, and other immune cells and participates in the occurrence and development of immune diseases. Zhang et al. found that the secretion of TL1A promotes the occurrence of rheumatoid arthritis; inflammatory cytokines also stimulate the secretion of TL1A to form a vicious cycle, which exacerbates the pathogenesis of rheumatoid arthritis [29]. Li et al. reported that the activation of DR3 signaling increases GM-CSF production via the p38 MAPK pathway, leading to ILC3 loss from the intestine and exacerbating colitis [30]. The current study confirmed that the TL1A/DR3 axis is involved in the airway remodeling process in asthma [17, 31]. However, both TL1A KO or CKO and DR3 CKO result in severe inflammation and lung edema after LPS stimulation in mice, further confirming that intact TL1A/DR3 signaling is beneficial for the maintenance of the lung epithelial barrier. This anti-inflammatory and protective effect is in contrast to the proinflammatory and damaging effect of TL1A/DR3 in various chronic diseases. This functional "paradox" of TL1A/DR3 is likely to be related to the different types of inflammatory cells or inflammatory mechanisms involved in different diseases. ARDS involves the transient disruption of the epithelial barrier, which can be spontaneously repaired or eventually progress to fibrosis [32]. The current results support the findings of Jia et al. [19], Yang et al. [20], Li et al. [21], and Shimodaira et al. [33]. Therefore, the TL1A/DR3 axis can be regarded as a double-edged sword, which is of great significance in guiding the clinical application of TL1A drugs.

The cathepsin family is huge (from cathepsin A to cathepsin Z); most of its members belong to cysteine proteases, cathepsin D and E are aspartic proteases, and cathepsin A and G are serine proteases. Cathepsin mainly exists in immune cells but is also detected in gastric mucosal and lung epithelial cells [34, 35]. Correlations between cathepsin E and lung diseases have been reported [13, 14]. In addition, Ctse in liver ischemia and reperfusion injury regulates macrophages and innate immunity [36]. However, little is known about the role of Ctse in lung injury. In the present study, TL1A-KO significantly increased Ctse expression and activity in mice with LPS-induced ARDS. TL1A-CKO also significantly increased Ctse expression in alveolar epithelial cells from the mouse model of LPS-induced ARDS. Similar results were obtained in DR3-CKO mice with LPS-induced ARDS. Hou et al. revealed that glycoprotein 5 is cleaved by Ctse during porcine reproductive and respiratory syndrome virus membrane fusion [37]. The glycocalyx is a villous polyglycoprotein complex structure located in the outer membrane of cells. Our results further confirmed that Ctse promotes the progression of inflammation in LPS-induced ARDS and the destruction of the glycocalyx, as determined by overexpressing Ctse in mouse alveolar epithelial cells. In vitro, the expression of Ctse was inhibited after LPS stimulation in hPAEPICs overexpressing TL1A and DR3, while SDC-1 shedding was reduced. SDC-1 remarkably decreased after stimulating hPAEPICs with recombinant human Ctse. Cathepsin L activates HPA, which can induce the removal of heparan sulfate and SDC-1 [38]. In addition, by using a proteomics approach, the results showed that TL1A KO attenuated MMP-9 expression in an LPS-induced ARDS model. These results are consistent with those of Yang, in which VEGF-induced increase in MMP-9 in peripheral blood vessels was inhibited in TL1A-transgenic mice after intracerebral hemorrhage [20]. The differences in results could be explained by differences in the mouse disease model and the distribution of TL1A in mice.

The glycocalyx, as a natural barrier on the cell surface, is easily affected by shear force, enzymes, oxidative stress, and inflammation and is in a dynamic state [39, 40]. Glycocalyx is critical for blood–brain barrier integrity by suppressing caveolin1-dependent endothelial transcytosis following ischemic stroke [41]. Notably, the glycocalyx has additional functions in addition to this single barrier function. Hyaluronan synthase 2-mediated glycocalyx hyaluronan (HA) production mediates liver fibrosis [42]. In addition, smooth muscle cell-derived HA modulates the vascular SMC-phenotype in murine atherosclerosis [43]. Therefore, the glycocalyx has a barrier function in the early stage of ARDS and exerts a protective effect. The present study confirmed that TL1A/DR3 CKO aggravates glycocalyx damage in the early stage of ARDS. Moreover, the TL1A/DR3 axis is a key protective factor in the early stage of ARDS.

Lung epithelial tight junctions have been studied extensively in research on cell permeation and edema [44]. In addition, the modulation of tight junctions by absorption enhancers can improve paracellular drug transport and hence drug delivery [45]. However, the functions of Tjp-3 depend on the conditions. The exogenous expression of the amino-terminal portion of Tjp-3 negatively affects the assembly of tight junctions and adhesive junctions [46]. Li et al. indicated that Tjp-3 plays a vital role in the protection of intestinal epithelial barrier function under inflammatory conditions [47]. Kiener et al. also found that Tjp-3 is critical for epidermal barrier function in zebrafish embryos [48]. In the present study, TL1A/DR3 CKO modulated the LPS-induced tight junctions of alveolar epithelial cells in mice. Further in vitro and in vivo analyses confirmed that TL1A/DR3 affected Tjp-3 by regulating the expression and activity of Ctse. These results improve our understanding of the regulatory mechanism and function of Tjp-3 in ARDS.

Mainstream viral vectors mainly consist of LV, adenoviruses, and AAV. Among these, AAV benefits from its general lack of pathogenicity and is widely used in basic research and clinical trials [49]. Nahmad et al. used two AAVs to simultaneously deliver CRISPR-Cas9 and immunoglobulin gene repair templates, directly causing B cells to produce neutralizing antibodies against HIV, resulting in application prospects in HIV treatment [50]. In the present study, AAVs were used for the epithelial cell-specific deletion of TL1A and OE of Ctse in mice, providing a solid experimental and theoretical foundation for future clinical trials.

## Conclusions

In conclusion, a novel protective role of TL1A/DR3 in ARDS was demonstrated. These results point to a central role for TL1A/DR3 signaling in barrier formation in alveolar epithelial cells and suggest that these effects are mediated by inflammatory signaling and Ctse expression. This study lays a pharmacological foundation for TL1A/DR3 pathway drugs.

## Supplementary Information

**Additional file 1.** hPAEPIC activity at different concentrations of LPS was detected by cell counting kit 8 (CCK-8) assay.

## Data Availability

The raw data of *Mus musculus* and hPAEPICs mRNA-sequencing were deposited to NCBI under the BioProject ID: PRJNA930203 and PRJNA930588, respectively. The raw data of *M. musculus* single cell RNA-sequencing were deposited to NCBI under the BioProject ID: PRJNA931725. The raw data of proteomic analysis were deposited to ProteomeXchange under the Project ID: IPX0005861001. Accession numbers are listed in the key resources table. This study does not report the original code. Any additional information required to reanalyze the data reported in this paper is available from the lead contact upon request.

## Abbreviations

KO: Knockout
Ctse: Cathepsin E
HPA: Heparanase
OE: Overexpression
SDC-1: Syndecan-1
AT II: Alveolar type II
DR3: Death receptor 3
LPS: Lipopolysaccharide
MMPs: Matrix metalloproteinases
ARDS: Acute respiratory distress syndrome
MLE-12: Cells mouse alveolar epithelial cells
CKO: Alveolar epithelium conditional knockout
Tjp-3: Tight junction-associated zonula occludens 3
hPAEPICs: Human pulmonary alveolar epithelial cells
TL1A: Tumor necrosis factor ligand-associated molecule 1A
