# Supplementary material for: Identification of a novel role for TL1A/DR3 deficiency in acute respiratory distress syndrome that exacerbates alveolar epithelial disruption
Source: Respir Res. 2023 Jul 11;24:182. doi: 10.1186/s12931-023-02488-1 (PMC10334539; doi:10.1186/s12931-023-02488-1)
Supplement: Supplementary file 1 — Additional file 1. hPAEPIC activity at different concentrations of LPS was detected by cell counting kit 8 (CCK-8) assay. [file 12931_2023_2488_MOESM1_ESM.docx]

**Method**

**Cell activity assay**

hPAEPIC activity was detected by cell counting kit 8 (CCK-8) asssy (C0037, Beyotime Biotechnology). HPAEpiCs were added to sterile 96-well plates and incubated with LPS of different concentrations (0, 1, 5, 10, 20 μg/mL) for 6 h. Then an equal amount of CCK-8 reagent was added to each well and cultured with hPAEPICs for 2 h. Lastly, 96-well plates were measured by a microplate reader at 450 nm.

**Figure S1**


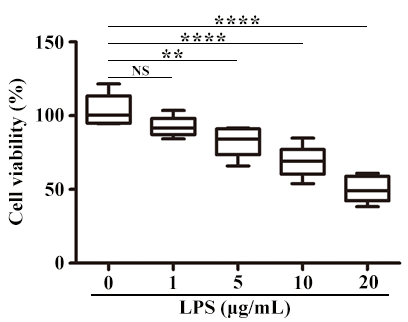


**Figure S1 Legends**

hPAEPICs were incubated with different concentrations of LPS (0, 1, 5, 10, 20 μg/mL). hPAEPICs viability was detected by CCK-8 assay. Data are expressed as the means ± SD of three independent experiments. *p < 0.05, **p < 0.01, ***p < 0.001, ****p < 0.0001. NS, not significant.
